# PARTNERS IN CARE MODEL FOR PERSONS WITH DEMENTIA AND MULTIMORBIDITY (PCM)

**DOI:** 10.1093/geroni/igae098.4041

**Published:** 2024-12-31

**Authors:** Ingelin Testad, Martha Gjestsen, Lise Birgitte Holteng

**Affiliations:** Centre for Age-Related Medicine - SESAM, Stavanger University Hospital, Stavanger, Rogaland, Norway; Stavanger University Hospital, Stavanger, Rogaland, Norway; Stavanger University Hospital, Stavanger, Rogaland, Norway

## Abstract

The increasing prevalence of old age, multimorbidity and dementia over time suggest a pressing need for interventions which can help mitigate the health challenges associated with an aging population. One of the challenges adhere to the complexity and frequency of interactions with- and transitions between multiple health services, which further increases the vulnerability to coordination breakdowns, thus representing organisational challenges for the health system as well as to the care of this vulnerable group of people. This project represents a development of a Partner in Care Model (PCM) to meet the challenges persons with multimorbidity and dementia are met with. The Partner in Care Model will organize the care within the existing health care system, to increase the health care professionals expertise in involving carers to give high quality and cost effective care to persons with multimorbidity and dementia, to meet the major challenges this vulnerable group of people. Furthermore, PCM will have a holistic approach to meet all the challenges in this vulnerable group of people, on the management of medical-, lifestyle and psychosocial-health care needs. More specifically we aim to: • Identify the most common care models used in managing persons with multimorbidity with dementia • Describe the effectiveness of existing care models to develop a Partner in Care Model • Test the feasibility of a Partner in Care Model

